# Effects of Applying Organic Amendments on Soil Aggregate Structure and Tomato Yield in Facility Agriculture

**DOI:** 10.3390/plants13213064

**Published:** 2024-10-31

**Authors:** Wen-Qu Tao, Qian-Qian Wu, Jie Zhang, Ting-Ting Chang, Xin-Na Liu

**Affiliations:** College of Agricultural Science and Engineering, Hohai University, Nanjing 210098, China; tao18955688309@163.com (W.-Q.T.); doris_wqq@163.com (Q.-Q.W.); changtt@hhu.edu.cn (T.-T.C.); 18262627128@163.com (X.-N.L.)

**Keywords:** biochar, vermicompost, mineral-source potassium fulvic acid, aggregate stability, nutrient

## Abstract

Amendment significantly improves soil structure and promotes crop growth. To combat soil degradation and low crop yields in facility agriculture, it is crucial to study the optimal application rate of amendments. This study analyzed the effects of biochar, vermicompost, and mineral-source potassium fulvic acid on the stability of aggregate structure, soil nutrient content, and tomato yield in cambisols, providing a theoretical basis for improving the soil quality of plastic greenhouses in Southern China. A pot experiment on tomato cultivation was carried out in yellow-brown soil in plastic greenhouses. The experiment included eight treatments: 1% biochar (B1); 3% biochar (B3); 5% biochar (B5); 3% vermicompost (V3); 5% vermicompost (V5); 0.1% mineral-source potassium fulvic acid (F1); 0.2% mineral-source potassium fulvic acid (F2); and the control condition without adding soil amendments (CK). The results showed that the biochar and vermicompost treatments effectively reduced soil bulk density and increased total soil porosity. Compared to the control, treatments with soil amendments significantly increased soil pH and had different effects on soil nutrients: F2 showed the most significant improvement in the content of available nitrogen, available phosphorus, and available potassium, with an increase of 133.33%, 834.59%, and 74.34%, respectively; B3 treatment had the highest increase in dissolved organic carbon (DOC), while B5 treatment had the highest organic matter content. Compared to the CK, the particle size of the biochar treatment was mainly 0.053~0.25 mm, while the V3, F1, and F2 mainly occurred with a particle size > 0.25 mm; and V3 has the best aggregate stability. Biochar, vermicompost, and mineral potassium fulvic acid can all promote tomato yield, with the F2 and V3 treatments having a yield increase effect of over 30%. Furthermore, Pearson’s correlation analysis showed a highly significant positive correlation between geometric mean diameter (GMD) and mean weight diameter (MWD), water-stable macroaggregate content (R_0.25_), and a positive correlation between alkaline-dissolved nitrogen, available phosphorus, dissolved organic carbon content, and aggregate stability indicators. Adding 0.2% mineral-source potassium fulvic acid optimizes cambisols’ properties, enhances aggregate formation and stability, boosts tomato yield, and shows great application potential.

## 1. Introduction

In China’s facility-based agriculture, soil cultivation remains the predominant method, making the quality of facility soil a key factor limiting the sustainable development of both current and future agricultural practices. Within the ecosystem, soil serves as a crucial hub for material exchange and energy transfer, with its structure influencing water retention, thermal regulation, and nutrient availability, which directly impact crop yield and quality [1]. However, the excessive use of chemical fertilizers and pesticides, high multiple cropping indexes, and long-term monocultures have led to widespread soil degradation in facility cultivation, significantly reducing crop productivity [2,3].

Currently, soil amendments play a vital role in enhancing soil fertility and structure by improving its physical properties, increasing water retention capacity, and enhancing the availability of essential nutrients [4,5]. By fostering better soil aeration, permeability, and aggregation, soil amendments contribute to a more favorable environment for root growth [6] and microbial activity [7]. This, in turn, facilitates nutrient cycling, enhances soil fertility, and promotes the cultivation of healthier crops [8,9]. As such, soil amendments represent a critical tool in mitigating soil degradation and supporting the long-term sustainability of agricultural systems, particularly in facility-based agriculture where soil quality is a limiting factor.

Numerous studies have demonstrated that the application of biochar, vermicompost, and mineral-sourced potassium fulvic acid can significantly improve soil quality and promote crop yield [10,11,12,13,14]. Biochar, an organic amendment produced through the pyrolysis of biomass at high temperatures, possesses a large specific surface area and highly porous physical structure [15]. These characteristics enhance the soil’s water retention capacity and contribute to the formation of larger soil aggregates [16]. Additionally, biochar has a high cation-exchange capacity and strong adsorption properties, providing a comprehensive range of nutrients that support the improvement of soil fertility [17]. Research has indicated that higher application rates of biochar are more effective in enhancing the structure of cambisols, while lower application rates maximize nutrient availability in the soil [18]. Vermicompost, a nutrient-rich organic fertilizer, offers excellent aeration, drainage, and microbial activity, facilitating efficient nutrient uptake by crops [19]. Liu et al. found that the combination of vermicompost and humic acid fertilizer effectively improved the stability of soil aggregates and enhanced the microstructure of larger aggregates while increasing the soil’s organic matter content [20]. Fulvic acid is the water-soluble part of humic acid, and humic acid contains fulvic acid [21]. Mineral-sourced potassium fulvic acid, an environmentally friendly soil amendment, not only improves soil structure and fertility [22] but also enhances crop resilience to environmental stress [23]. Fulvic acid ensures the effective absorption of nutrients by plants [24], and the application of potassium fulvic acid in Southern China has been shown to significantly improve both crop yield and quality [25].

Currently, most studies on the use of biochar, vermicompost, and mineral-sourced potassium fulvic acid have focused on their individual application or combined use in fixed proportions, with few studies examining their comparative effects on facility soil [26,27,28,29]. To identify the organic amendment with the most comprehensive improvement effect on facility soils in Southern China, this study utilized yellow-brown soil from the middle- and lower-reaches of the Yangtze River as the test soil. Through a pot experiment involving facility-grown tomatoes, this study investigated the effects of adding biochar, vermicompost, and mineral-sourced potassium fulvic acid on soil nutrient content, soil aggregate structure, and tomato yield. The findings provide a theoretical basis for enhancing soil quality and crop productivity in facility-based agriculture.

## 2. Results

### 2.1. Effects of Different Amendments on Soil Chemical Properties

**Figure 1:**
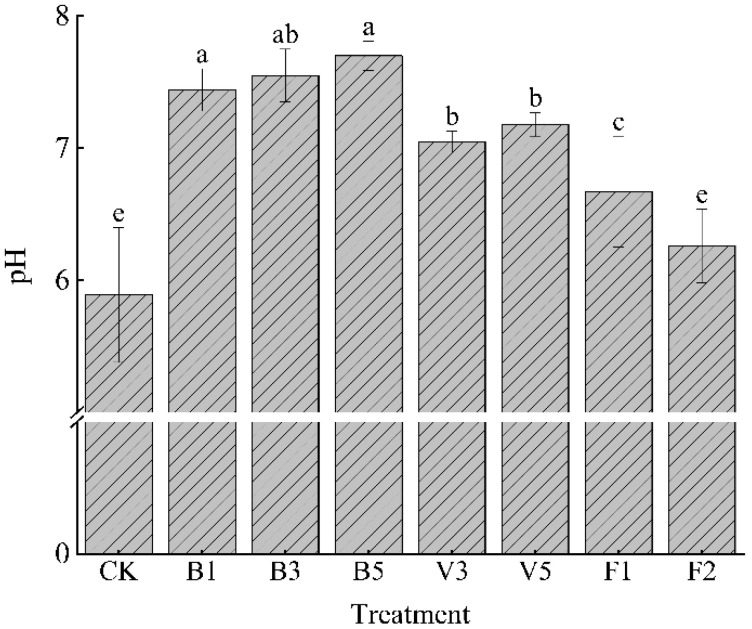
Effects of different soil amendments on soil pH. Note: CK: control condition, B1: 1% biochar, B3: 3% biochar, B5: 5% biochar, V3: 3% vermicompost, V5: 5% vermicompost, F1: 0.1% mineral-source potassium fulvic acid, F2: 0.2% mineral-source potassium fulvic acid; the means are not significantly different between soil type treatment (*p* > 0.05) when followed by the same lowercase letter, according to Duncan’s multiple range test; ANOVA, analysis of variance.

After applying different amendments, the soil chemical properties of each treatment changed. As shown in Figure 1, the pH values of each treatment were significantly higher than the CK; the improvement effect of biochar treatment was more obvious than that of the vermicompost treatment and mineral potassium fulvic acid treatment, and the pH value of B5 was the highest (7.7). As can be seen from Table 1, biochar treatment significantly increased the soil-available potassium and organic matter contents compared with the CK, and the contents were positively correlated with the applied amount of biochar. The contents of alkali-hydrolyzed nitrogen, available phosphorus, and DOC were higher than those in the treatment without amendment, and B3 content was the highest, indicating that the high biochar application rate was more conducive to promoting the absorption of available nutrients in soil. The nutrient index content of V5 was significantly higher than that without amendment (*p* < 0.05). The contents of V3 available potassium, organic matter, and DOC were the lowest values in the treatment of adding amendments, which indicated that the application of 3% vermicompost had a poor effect on the soil nutrient content. In the treatment of potassium fulvate from a mineral source, the contents of alkali-hydrolyzed nitrogen, available phosphorus, and available potassium in F2 were the highest values in the treatment of adding amendments, and the increases were 133.33%, 834.59%, and 74.34% compared with the CK, respectively. The effect of promoting soil absorption of nitrogen, phosphorus, and potassium is the most obvious.

### 2.2. Effects of Different Amendments on Soil Structure

#### 2.2.1. Effects on Soil Bulk Density and Total Porosity

**Figure 2:**
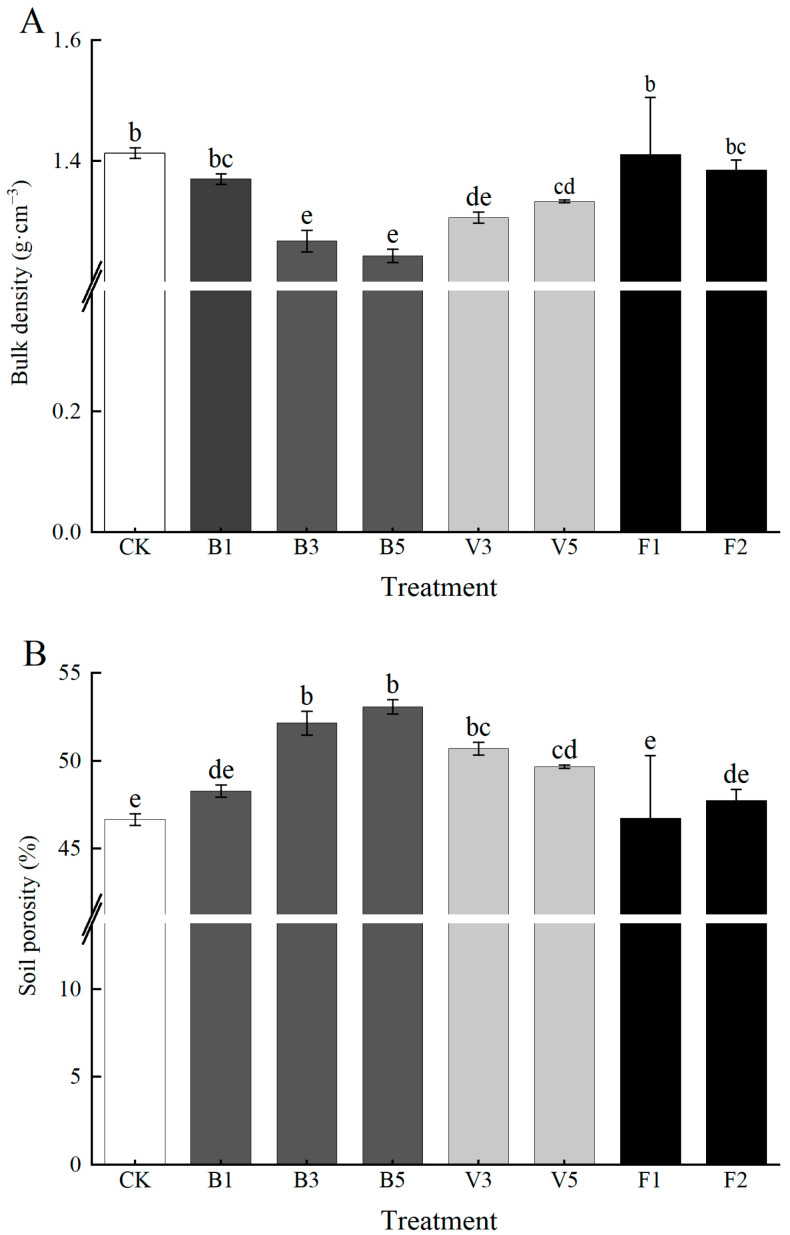
(**A**,**B**) Effects of different soil amendments on soil bulk density and porosity. Note: CK: control condition, B1: 1% biochar, B3: 3% biochar, B5: 5% biochar, V3: 3% vermicompost, V5: 5% vermicompost, F1: 0.1% mineral-source potassium fulvic acid, F2: 0.2% mineral-source potassium fulvic acid. The means are not significantly different between soil type treatment (*p* > 0.05) when followed by the same lowercase letter, according to Duncan’s multiple range test; ANOVA, analysis of variance.

Soil bulk density is one of the important indexes of soil quality. Compared with the CK, the changes in soil bulk density were different under the action of different amendments. With the increase in biochar application, soil bulk density showed an obvious decreasing trend, in which B5 had the most obvious decreasing effect, reaching 12.06%. Compared with the CK, V3 and V5 decreased by 7.09% and 5.67%, respectively. The difference between potassium fulvic acid treatment and the CK is not significant, and the effect of reducing soil bulk density is not obvious. As one of the important parameters of soil structure, soil total porosity reflects soil permeability. As shown in Figure 2B, biochar and vermicompost treatments significantly increased the total soil porosity compared with the CK. B5 increased the total porosity of soil by 13.73%, and B1 and B3 increased by 3.45% and 11.76% compared with the CK, respectively. The effect of vermicompost application on total soil porosity decreased with the increase in vermicompost application amount, and V3 and V5 increased by 8.64% and 6.45%, respectively. F1 and F2 only increased by 0.17% and 2.29%, respectively. The results showed that B5 had the most significant effect on soil bulk density and total porosity.

#### 2.2.2. Effects of Different Soil Amendments on Soil Texture

**Figure 3:**
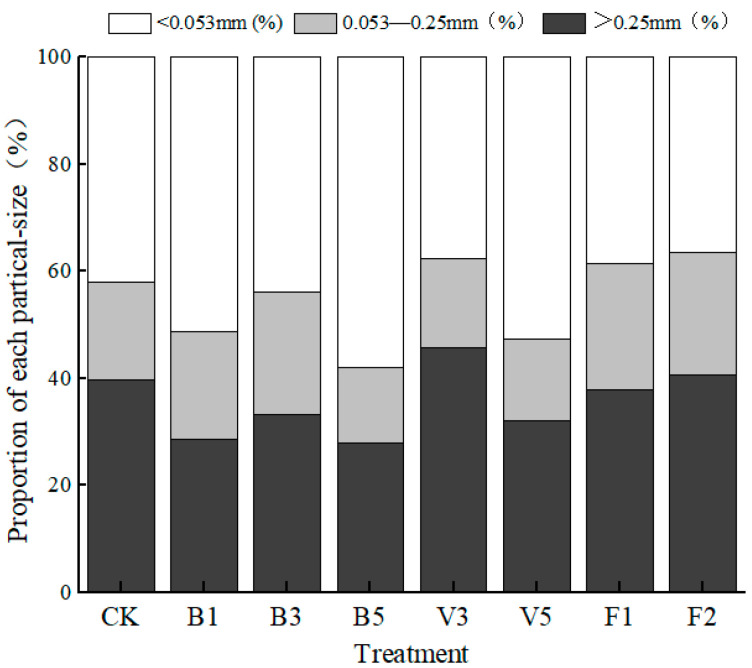
Fractions of soil aggregates under different soil amendments. Note: CK: control condition, B1: 1% biochar, B3: 3% biochar, B5: 5% biochar, V3: 3% vermicompost, V5: 5% vermicompost, F1: 0.1% mineral-source potassium fulvic acid, F2: 0.2% mineral-source potassium fulvic acid.

Soil aggregate is the basic unit of soil structure and the particle size distribution of aggregates is affected by the application of amendments. As can be seen from Figure 3, the content of the CK macroaggregates (particle size > 0.25 mm) is roughly the same as that of silky clay particles (particle size < 0.053 mm), which are 39.71% and 42.13%, respectively. Compared with the CK, the distribution of soil aggregates changed significantly in the treatment group, and the effects of different soil amendments were also different. Biochar treatment obviously increased the content of silt clay and decreased the content of macroaggregates. The content of B5 powder clay is the highest in all treatments (57.97%), with an increase of 37.60%; the content of B3 macroaggregates was the highest in the biochar treatment (33.35%). There were significant differences in the powder clay content of vermicompost treatment. Compared with the CK, the content of V3 decreased by 10.56%, while that of V5 increased by 24.78%. The powder clay content of F1 and F2 decreased by 8.33% and 13.17% compared with the CK, respectively. The contents of microaggregates (0.053~0.25 mm) increased significantly, which were 23.60% and 22.84%, respectively.

#### 2.2.3. Effects of Different Soil Amendments on Soil Aggregation Parameters

**Figure 4:**
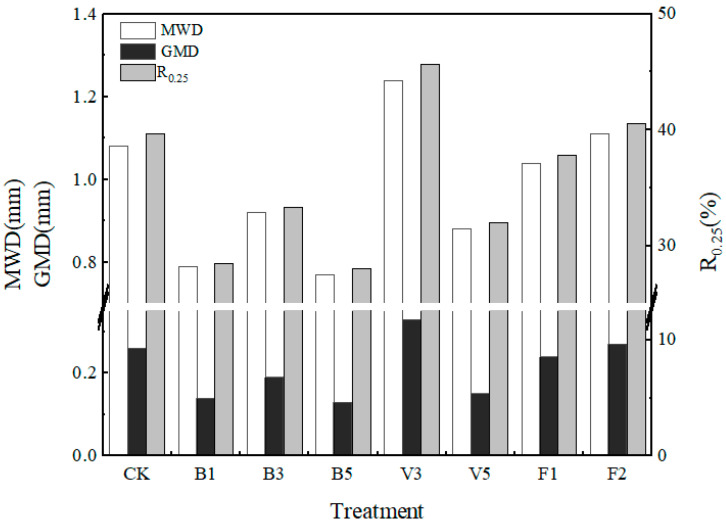
Stability indicators of soil aggregates under different soil amendments. Note: CK: control condition, B1: 1% biochar, B3: 3% biochar, B5: 5% biochar, V3: 3% vermicompost, V5: 5% vermicompost, F1: 0.1% mineral-source potassium fulvic acid, F2: 0.2% mineral-source potassium fulvic acid.

The aggregate mean weight diameter (MWD), geometric mean diameter (GMD), and water stability of large aggregate content (R_0.25_) are important indexes to characterize the stability of soil aggregates. The higher the value, the better the stability of soil aggregates. As shown in Figure 4, all stability indexes of biochar treatment are presented in the order of B3 > B1 > B5, in which all the stability indexes of B5 are the lowest values in all treatments. Compared with the CK, the stability indexes of V5 and F1 all decreased, and the MWD decreased by 18.52% and 3.70%, respectively; GMD decreased by 42.31% and 7.69%, respectively; and R_0.25_ decreased by 19.34% and 4.84%, respectively. Compared with the CK, the stability of V3 and F2 aggregates was significantly improved, among which the stability indexes of V3 were the highest, and MWD, GMD, and R_0.25_ were increased by 14.81%, 26.92%, and 15.08%, respectively, indicating that 3% vermicompost treatment had the best effect on the stability of soil aggregates.

### 2.3. Effects of Different Amendments on Tomato Yield

**Figure 5:**
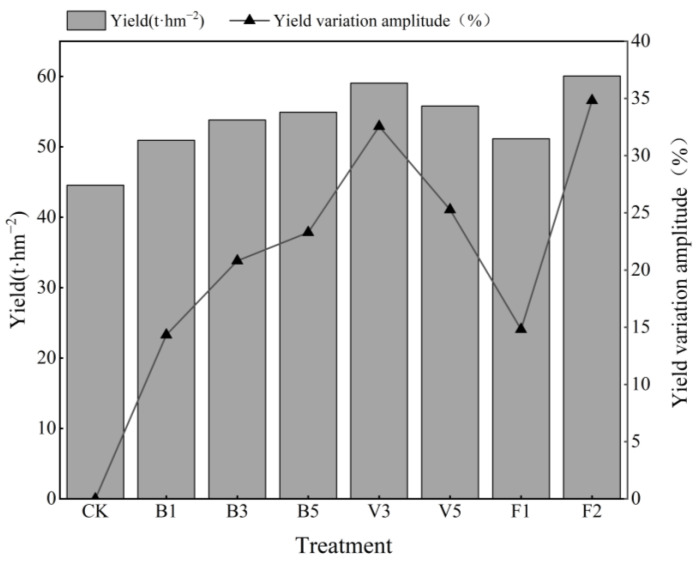
Effects of different soil amendments on yield. Note: CK: control condition; B1: 1% biochar; B3: 3% biochar; B5: 5% biochar; V3: 3% vermicompost; V5: 5% vermicompost; F1: 0.1% fulvic acid; F2: 0.2% fulvic acid. The magnitude of yield change was calculated using the control group as a reference.

Yield is a key indicator of crop production. After the application of the soil amendment, the yield of each treatment increased compared with the CK (as shown in Figure 5), and the increase effect of V3 and F2 reached more than 30%. The yield of biochar treatment increased with the increase in application amount, and were 50.92, 53.81, and 54.91 t·hm^−2^, respectively. Compared with the CK, vermicompost treatment increased yield by 32.56% (V3) and 25.28% (V5), respectively. Under the same application amount, the yield of the vermicompost treatment was higher than that of biochar treatment. F2 increased the most, reaching 34.82%, and had the best effect on yield increase.

### 2.4. Correlation Analysis

**Figure 6:**
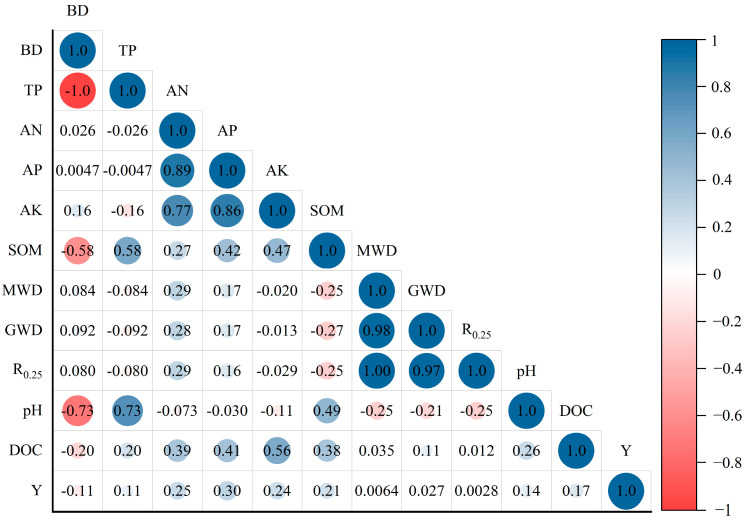
Heat map for correlation analysis of soil indicators. Note: BD: bulk density; TP: total porosity; AN: available-N; AP: available-P; AK: available-K; SOM: soil organic matter; MWD: mean weight diameter; GWD: geometric mean diameter; R_0.25_: water-stable large aggregate content; DOC: dissolved organic carbon; Y: yield. The numbers in the figure represent correlation coefficients, with a significance level of *p* < 0.05.

The bulk density is significantly negatively correlated with total porosity and organic matter. In Figure 6, it can be seen that the physical structure of the soil affects the uptake of organic matter by the soil, which is consistent with the facts. The looser the structure, the more conducive it is to the increase in organic matter content in the soil. Soil chemical indexes were closely related, and there were significant positive correlations among alkali-hydrolytic nitrogen, available phosphorus, and available potassium. pH had certain effects on soil nutrient content and was negatively correlated with both of them. There was a significant negative correlation between organic matter and pH as well as between available phosphorus and available potassium. The indexes of soil aggregates reflect the anti-erosion ability of the soil structure. Aggregate parameters, available nutrients, and organic matter content were positively correlated with yield, indicating that the increase in soil nutrient content and aggregate stability was conducive to the increase in tomato yield.

### 2.5. Gray Correlation Analysis of Parameters Affecting the Effectiveness of Soil Improvement

In this experiment, a total of 11 soil parameters and 8 treatments were selected, including total porosity (TP), average weight diameter of aggregates (MWD), geometric mean diameter (GMD), water-stable large aggregates content (R_0.25_), pH value, dissolved organic carbon content (DOC), soil organic matter (SOM), alkaline-dissolved nitrogen content (AN), quick-acting phosphorus content (AP), quick-acting potassium content (AK), and yield (Y) (Table 2). The weighted gray correlation was calculated and the best value of each indicator was used as a reference series. When evaluating soil quality, since there is no uniform standard for the selection of indicators, the use of gray correlation analysis can make the evaluation results more accurate and objective.

The differences in the effects of the treatments on soil nutrients, aggregate structure, and tomato yield enhancement have enabled a better evaluation of the effects of biochar, vermicompost, and mineral-source potassium fulvic acid. A total of 11 parameters and 8 treatments were employed to construct a gray system, with the optimal value of each index—listed above in this section—utilized as a reference series for the calculations. The greater the total weighted correlation degree, the better the soil improvement effect. As can be seen from Table 3, the comprehensive analysis results of the gray correlation degree in this test are F2 > V3 > B3 > B5 > B1 > F1 > CK > B1. The results showed that the comprehensive performance of 0.2% mineral-source potassium fulvic acid in this experiment is the best.

## 3. Discussion

Soil bulk weight—as an important factor in the physical structure of the soil—is closely related to the total soil porosity [30] and is significantly affected by the application of organic materials [31,32]. It is generally believed that soil pore plugging will lead to soil compaction, which will affect the water, fertilizer, gas, and heat status of the soil [33]. Previous studies have concluded that the lower the value of soil bulk density, the greater the total porosity, which is conducive to reducing soil consolidation and promoting the formation of soil aggregates [34,35]. The results of this paper are in line with previous research. A good aggregate structure distribution is conducive to soil material exchange and energy transfer and promotes soil water and fertilizer retention and carbon sequestration [36,37]. In this experiment, both the bulk density and total porosity of the soil treated with biochar showed a trend of gradually improving with the increase in the amount of biochar (Figure 2). Due to its high specific surface area and porosity, biochar, when applied as an amendment to the soil, improves soil properties, resulting in an increase in soil bulk density and total porosity [38,39]. However, the content of water-stable macroaggregates decreased compared with the CK, which may be due to changes in exchange complexes caused by the application of biochar [40]. After the application of vermicompost, the changes in soil bulk density and total porosity were between those of biochar and the potassium fulvic acid treatment. Under the same application amount, the soil bulk density value of vermicompost treatment was higher than that of the biochar treatment, and showed a trend of change opposite to that of the biochar treatment, possibly because the physical structure of the vermicompost used in this study was too tight [41]. Compared with biochar and vermicompost, the changes in bulk density and total porosity under potassium fulvic acid treatment from mineral sources are not obvious, which may be due to the fact that the application amount of potassium fulvic acid from mineral sources was potentially too small [42].

The indexes of soil aggregates reflect the anti-erosion ability of soil structure. The larger the mean geometric diameter and geometric weight diameter, the higher the soil agglomeration degree and the better the anti-erosion ability [43]. A high proportion of water stability and large aggregate content will be more conducive to soil fertility, thereby improving soil quality. In this experiment, all the indexes of B5 were the lowest values in all treatments, indicating that the high application amount of biochar was not conducive to the parameters of the soil aggregate structure. The parameters of the aggregates are consistent with the results of particle size distribution changes [44], and V3 has the best improvement effect on the stability index of aggregates compared with other treatments (Figure 4). It is generally believed that soil organic matter, as a cementing agent of soil aggregates, is conducive to the formation of large aggregates and the improvement of aggregate stability [45,46,47]; however, the content of organic matter is not the only factor affecting the composition of aggregates. In this study, the content of organic matter was negatively correlated with MWD, GMD, and R_0.25_ (Figure 6); this may be due to the wet sieve method used in the test [48] or the existence of exchangeable sodium, which would lead to the destruction of aggregates [45,48]. As an important part of soil, organic matter has an important influence on soil fertility and physical structure. Under the conditions of this test, the application of amendments significantly enhanced the soil organic matter content compared to the control. Additionally, for each amendment, an increase in application rate corresponded to a higher soil organic matter content. Notably, the amendment B5 exhibited the highest organic matter content, attributed to the elevated carbon content of biochar. Furthermore, the effects of the amendments on organic matter enhancement became more pronounced with increased application rates [49].

Soil-available nutrient content comprehensively reflects its fertilizer supply capacity, which has a key effect on tomato growth and yield. Biochar is rich in nitrogen, phosphorus, and potassium; vermicompost is rich in nutrition; mineral-source potassium fulvic acid contains a variety of micronutrients; and the three soil amendments can improve the physical and chemical properties of soil [14,50,51]. The study by Li Zhipeng et al. showed that compared with other treatments, the application of fertilizer combined with 1800 kg·hm^−2^ mineral-source potassium fulvate had the best effect on increasing the content of soil-available nutrients and improving soil fertility [52]. In this study, the contents of alkali-hydrolyzed nitrogen, available phosphorus, and available potassium in the F2 treatment were the highest among all treatments (Table 1). On the one hand, the mineral-source potassium fulvic acid itself contains rich potassium elements, which is conducive to soil absorption; on the other hand, it may also be due to the oxygen-containing functional groups of the potassium fulvic acid from mineral sources, which play an active role in the activation of nutrients fixed in the soil and the secretion produced by the roots of planted crops—promoting the release of nutrients [53]. Compared with potassium fulvic acid treatment from mineral sources, biochar and vermicompost treatment had a less obvious effect on nutrient content, which may be due to the absorption of available nutrients by biochar and vermicompost [54] or the chemical fixation reaction of clay minerals in the amendment [55]. Soil amendments can significantly promote the improvement of crop yield [56,57]. In this experiment, 0.2% mineral-source potassium fulvic acid had a significant effect on tomato yield increase, which was consistent with the results of Li Jing et al. [58], further confirming that rational application of mineral-source potassium fulvic acid could not only promote nutrient accumulation but also improve crop yield.

## 4. Materials and Methods

### 4.1. Experimental Materials

The tested biochar was corn stalk carbon, purchased from Henan Lize Environmental Protection Technology Co., Ltd. (Henan, China). Its main physical and chemical properties are as follows: pH 9.40; organic carbon 410.90 g·kg^−1^; P_2_O_5_ 5.33 g·kg^−1^; K_2_O 19.16 g·kg^−1^; total nitrogen 8.35 g·kg^−1^; total phosphorus 2.33 g·kg^−1^; total potassium 15.90 g·kg^−1^.

The main physical and chemical properties of the tested vermicompost are as follows: pH 8.17; organic carbon 419.22 g·kg^−1^; total nitrogen 12.19 g·kg^−1^; total phosphorus 28.00 g·kg^−1^; total potassium 13.02 g·kg^−1^.

The tested biochar was obtained by firing at a constant temperature of 300 °C without oxygen for 2 h. The tested mineral-source potassium fulvic acid was purchased from Handan Saifu Biotechnology Co., Ltd. (Handan, China). The content of fulvic acid was ≥50%, the content of potassium oxide was ≥12%, and the content of humic acid was ≥60%.

### 4.2. Experimental Site Description

The experiment was carried out in plastic greenhouses (31°57′ N, 118°50′ E) in the Water-saving Park of Jiangning Campus of Hohai University, which exists within a subtropical monsoon climate with an average annual temperature of 15.7 °C, average annual precipitation of 1072.9 mm, and average annual evaporation of 900 mm. The cultivated soil was yellow-brown soil, and the basic properties of the soil before the test were as follows: pH 6.42; available phosphorus 5.81 mg·kg^−1^; available potassium 101 mg·kg^−1^; alkali-soluble nitrogen 11.10 mg·kg^−1^; organic matter 12.56 g·kg^−1^; total nitrogen 1.121 g·kg^−1^.

### 4.3. Experimental Design and Treatment Application

The trial was conducted from July to December 2020. A total of 8 soil amendment treatments were set up, namely: (1) 1% biochar (B1); (2) 3% biochar (B3); (3) 5% biochar (B5); (4) 3% vermicompost (V3); (5) 5% vermicompost (V5); (6) 0.1% mineral-source potassium fulvic acid (F1); (7) 0.2% mineral-source potassium fulvic acid (F2); (8) No soil conditioner (CK) was added. A total of 8 repetitions were set for each treatment.

Before the test began, the tested soil was air-dried and sieved through 2 mm, the amendment was thoroughly mixed with the soil, and then it was uniformly packed into a plastic bucket (diameter of 28 cm at the bottom; height of 38.5 cm). Each barrel contained 12 kg of soil and the bottom was filled with 8-centimeter-thick perlite. The bottom of the experimental bucket was perforated.

The tested tomato variety was “Cooperative 903” (cultivation density: 45,000 plants·hm^−2^), which was planted in plastic buckets at the late stage of the seedling stage, with 1 plant per barrel. Each barrel was applied with 20 grams of compound fertilizer (N:P:K = 15:15:15) as the base fertilizer. The plants retained 4 fruits per inflorescence, and the second inflorescence was pinked after the fruit.

### 4.4. Determination of Tomato Growth Indicators

During the growth period of the tomato plants, the plant height and stem diameter were measured by tape measure and vernier caliper every seven days. At the time of fruit ripening, the first and second inflorescence fruits of the selected plants were picked separately and the weight of individual fruits was measured and recorded using an electronic scale.

### 4.5. Determination of Soil Physical and Chemical Properties

After fruit harvest, three barrels were randomly selected for each treatment for soil sample collection as three repetitions. Soil samples from 5 to 10 cm were collected, removed, air-dried, and screened for the determination of the soil’s physical and chemical properties.

The ring knife method was used to determine soil bulk density and total porosity at 0~5 cm. The wet sieve method was used to analyze the structure of soil aggregates [59], The specific method is to place the soil sample on the shell sieve, add water to the settling bucket, wait until the water is over and infiltrated, and conduct oscillatory screening to separate the large aggregate (particle size > 0.25 mm), micro-aggregate (0.053–0.25 mm), and silky clay (<0.053 mm) components. Finally, it is washed into an aluminum box, dried, and weighed. The specific calculation formula of the soil aggregate stability index is as follows:

(1) $$MWD=\overset{n}{\underset{i=1}{\sum }}\bar{X_{i}}\omega_{i}\;$$

(2) $$GWD=EXP\left[\frac{\sum_{i=1}^{n}M_{i}\ln\bar{X_{i}}}{\sum_{i=1}^{n}M_{i}}\right]$$

(3) $$R_{0.25}=1-\frac{M_{<0.25}}{M_{T}}\times 100\%\;$$

where MWD is the average weight diameter (mm); $\bar{X_{i}}$ is the average diameter of particle *i* aggregate (mm); $\omega_{i}$ is the mass percentage (%) of the aggregate of grade *i*; GWD is the geometric mean diameter (mm); $M_{i}$ is the aggregate mass of *i* grade (g); $R_{0.25}$ is the content of water-stable macroaggregates (%); $M_{<0.25}$ is the mass sum (g) of aggregates with particle size < 0.25 mm; $M_{T}$ is the aggregate mass (g).

The dissolved organic content was determined in an extract with a soil–water ratio of 1:2.5 by shaking at 25.8 °C for 60 min and centrifugation at 4500 r/min for 10 min. The soil pH was measured in 1:5 soil and water extract using a calibrated pH meter. The available nitrogen was analyzed using the alkali-hydrolytic diffusion method [60]. The available phosphorus was determined by spectrophotometric method; the available potassium was determined by ammonium acetate extraction method; the organic matter was determined by potassium dichromate volumetric method.

### 4.6. Gray Relational Analysis Method

The gray correlation analysis method evaluates the correlation between curves by assessing the similarity of their geometric shapes over a sequence. It measures the relationship between time-series data by quantitatively analyzing trends in their development and changes. The method calculates the gray correlation degree between a reference series and various comparison series based on this geometric relationship. When the changes between two factors in the sample data are more divergent, their correlation degree is lower; conversely, a smaller difference in changes indicates a higher correlation degree. The results of soil improvement of each treatment were comprehensively evaluated by gray correlation analysis [61].

The following are the specific calculation methods.

Index weight calculation (entropy weight method):

(4) $$\omega_{j}=\frac{1-e_{j}}{\sum_{j=1}^{m}\left(1-e_{j}\right)}\;$$

where $e_{j}$ is the entropy value of the J item.

Gray relational degree analysis:

(5) $${x^{\prime }}_{ij}=\frac{x_{ij}}{x_{0j}}$$

(6) $${x^{\prime }}_{ij}=1-\frac{x_{ij}}{x_{0j}}\;$$

(7) $$\xi_{i}\left(k\right)==\frac{min\left(i\right)min\left(k\right)\left|X_{0}\left(k\right)-X_{i}\left(k\right)\right|+\rho max\left(i\right)max\left(k\right)\left|X_{0}\left(k\right)-X_{i}\left(k\right)\right|}{\left|X_{0}\left(k\right)-X_{i}\left(k\right)\right|+\rho max\left(i\right)max\left(k\right)\left|X_{0}\left(k\right)-X_{i}\left(k\right)\right|}$$

(8) $$\gamma_{i}=\sum_{k=1}^{n}W\left(k\right)\xi_{i}\left(k\right)$$

where k is the k index; i is the i data; ρ is the resolution coefficient, the value is 0.5. From the theory of gray correlation analysis, it is known that the reference series is the best in the evaluation of soil quality, so when the correlation between the evaluated indexes and the reference series is larger, it reflects better soil quality.

### 4.7. Statistical Analysis

Microsoft Excel 2010 software and IBM SPSS Statistics 26.0 software were used for data collation and analysis, and Origin2021 software was used for mapping. Univariate ANOVA and Pearson’s correlation analysis were compared using Duncan’s method (*p* < 0.05).

## 5. Conclusions

Organic carbon, vermicompost, and potassium fulvic acid from mineral sources have obvious effects on soil and tomato yield but the difference in species and application amount will affect the improvement effect. Biochar significantly increased the pH of yellow-brown soil and decreased the bulk density of soil; the application rate of 5% was the best. The application of 3% vermicompost has the most obvious effect on promoting the formation of large aggregates and improving the stability of aggregates in yellow-brown soil. Mineral-source potassium fulvic acid is more conducive to the absorption of soil-available nutrients. According to the gray correlation degree analysis, under the conditions of this study, the comprehensive improvement effect of 0.2% mineral-source potassium fulvate was significantly better than the other treatments and had a more balanced effect on improving yellow-brown soil structure, increasing nutrient content, and promoting tomato growth. All of the above methods of determination are based on Bao [62].

## Figures and Tables

**Table 1:** Effects of different soil amendments on soil nutrient contents.

Treatment	DOC (mg·L^−1^)	Available-N (mg·kg^−1^)	Available-P (mg·kg^−1^)	Available-K (mg·kg^−1^)	Organic Matter (g·kg^−1^)
CK	15.19 ± 0.07 b	44.67 ± 2.39 d	11.16 ± 2.19 c	158.5 ± 2.62 c	11.07 ± 0.57 d
B1	16.69 ± 0.40 ab	58.47 ± 3.41 bcd	39.29 ± 6.26 b	181.27 ± 6.86 bc	14.65 ± 0.33 c
B3	20.15 ± 1.37 a	59.23 ± 3.07 bcd	47.35 ± 7.32 b	193.15 ± 12.37 bc	19.57 ± 1.65 ab
B5	18.44 ± 2.02 ab	55.93 ± 1.27 cd	40.26 ± 1.17 b	203.05 ± 1.98 b	21.89 ± 1.92 a
V3	15.35 ± 2.19 b	68.02 ± 7.55 bc	45.63 ± 7.23 b	159.49 ± 5.24 c	14.51 ± 0.17 c
V5	17.25 ± 1.11 ab	78.97 ± 7.62 b	50.85 ± 8.04 b	203.05 ± 8.63 b	15.51 ± 0.09 c
F1	18.81 ± 1.45 ab	62.25 ± 17.61 bcd	45.32 ± 2.90 b	216.91 ± 9.08 b	16.23 ± 0.72 bc
F2	19.49 ± 0.36 ab	104.23 ± 10.80 a	104.30 ± 20.74 a	276.33 ± 24.73a	18.08 ± 1.69 bc

Note: CK: control condition; B1: 1% biochar; B3: 3% biochar; B5: 5% biochar; V3: 3% vermicompost; V5: 5% vermicompost; F1: 0.1% fulvic acid; F2: 0.2% fulvic acid. The data in the table are expressed as mean ± standard deviation, the means are not significantly different between soil type of treatment (*p* > 0.05) when followed by the same lowercase letter, according to Duncan’s multiple range test; ANOVA, analysis of variance.

**Table 2:** Index weight data.

Treatment	$e_{j}$	Weight
TP	0.957837328	0.0571
R_0.25_	0.927749695	0.0979
MWD	0.929261959	0.0959
GMD	0.882924764	0.1587
AN	0.897705925	0.1386
AP	0.932153402	0.0919
AK	0.898540221	0.1375
SOM	0.949682607	0.0682
pH	0.96895736	0.0421
DOC	0.957870079	0.0571
Y	0.95940454	0.0550

**Table 3:** Weighted correlation analysis of indicators.

Treatment	WGCD	WO
CK	0.423	7
B1	0.402	8
B3	0.517	3
B5	0.504	4
V3	0.658	2
V5	0.462	6
F1	0.492	5
F2	0.769	1

## Data Availability

The original contributions presented in the study are included in the article, further inquiries can be directed to the corresponding author.
